# ‘One region to control them all'- the surprising effectiveness of network control theory in predicting post-stroke recovery from aphasia

**DOI:** 10.3389/fncom.2022.943396

**Published:** 2022-08-10

**Authors:** Mariia Popova, Kayson Fakhar, Wilhelm Braun

**Affiliations:** Institute of Computational Neuroscience, University Medical Center Hamburg-Eppendorf, Hamburg, Germany

**Keywords:** aphasia, post-stroke recovery, network control, controllability, transcranial magnetic stimulation, inferior frontal gyrus, anterior insula

## Introduction

Aphasia is a neurological disorder, often caused by lesions after a stroke in language-related areas of the left hemisphere. It results in severe impairments of both verbal and written language production and comprehension (Damasio, 1992). One fundamental goal in aphasia research is to understand the detailed mechanisms underlying the complex processes of language recovery. An understanding of these mechanisms is crucial as it allows for an accurate prognosis for patients suffering from post-stroke aphasia. These patients form a very heterogeneous group with a large variability in treatment outcome. In a recent paper in *The Journal of Neuroscience*, Wilmskoetter et al. (2022) have made a step toward unraveling the neurobiological mechanisms underlying the recovery in patients with post-stroke aphasia.

## Network control theory

Wilmskoetter et al. (2022) used network control theory (NCT) to determine how NCT measures of language-related regions can predict recovery from aphasia after a stroke in the left hemisphere. NCT is based on the notion that the brain can be modeled as a dynamical system consisting of a complex interconnected network of nodes whose activity traverses a wide range of states to support cognition and behavior (Gu et al., 2015). “State” here means the coordinated pattern of brain activity at a given time point. Several intriguing questions arise when adopting such a perspective. For example, what role does the architecture of the network play in its dynamics? How do nodes influence one another, and is it possible to manipulate them to steer the network's current dynamical state to a target state? When applied to the brain, NCT aims to address such questions. First, previous work has shown that the healthy brain is *globally controllable*. This implies that it is theoretically possible to steer its dynamics to particular target states from any node, although this might be very difficult to do in practice (Gu et al., 2015). Second, it was shown that brain regions can be distinguished by their ability to drive the brain's state to a particular target state. In NCT terms, a region has high *average controllability* if it can navigate the brain to many easy-to-reach states and has high *modal controllability* if it can push it to hard-to-reach states. It was also shown that regions with high average controllability are densely connected to the rest of the network while regions with high modal controllability have sparse connectivity (Gu et al., 2015). These findings in healthy brains open new avenues for exploration in diseased brains. Namely, what is the impact of lesions on brain controllability? Is a damaged brain still globally controllable and if it is, to what extent can the recovery be predicted by the controllability of involved nodes?

## Controllability and aphasia recovery

**Figure 1:**
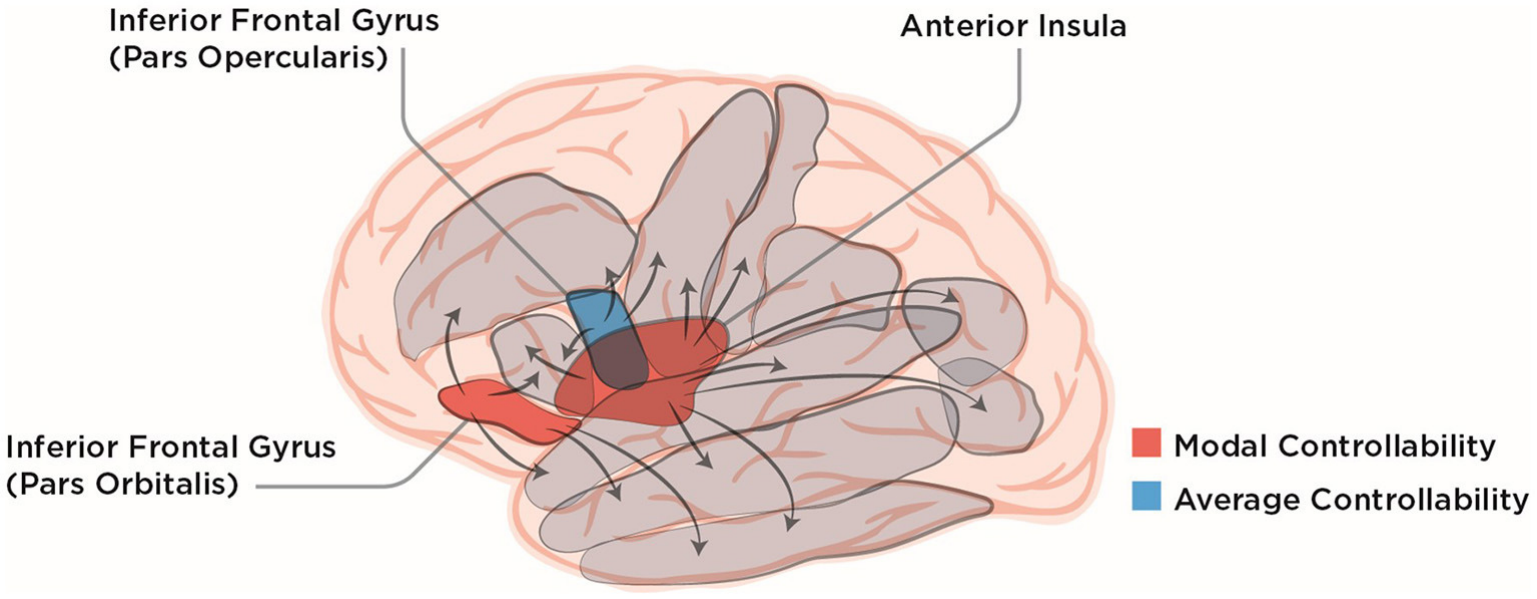
Lateral view of the human brain with ten of the language-related cortical regions chosen by Wilmskoetter et al. (2022) depicted in gray. IFG pars opercularis, pars orbitalis and the anterior Insula are colored according to their NCT controllability measures that were shown to reliably predict recovery from aphasia by Wilmskoetter et al. (2022). Arrows symbolically depict the influence brain regions can exert on each other which leads to different controllability values for different brain regions. The lateral view of the brain was modified from the work of Patrick J. Lynch, medical illustrator; C. Carl Jaffe, MD, cardiologist, under Creative Commons Attribution 2.5 License 2006 (https://commons.wikimedia.org/wiki/File:Brain_human_lateral_view.svg).

To explore what impact global, average, and modal controllability of the different brain regions have on the recovery from post-stroke aphasia, Wilmskoetter et al. (2022) used diffusion tensor imaging and probabilistic tractography. With that, they constructed connectomes of individuals suffering from post-stroke aphasia at the start of a three-week long language therapy. After that, they evaluated the therapy outcome by the correct responses in a standard naming test (Roach et al., 1996). Wilmskoetter et al. (2022) then chose 20 regions in the left hemisphere known to be involved in language understanding and speech production. Using a multiple linear regression model the authors found that, surprisingly, only a few control-theoretic measures applied to only a handful of specific brain regions were associated with treatment outcome (see Figure 1 for a schematic overview). In other words, the proper controllability measure had to be paired with the proper region to predict the naming score after therapy. Specifically, only average controllability of the inferior frontal gyrus (IFG) pars opercularis and modal controllability of the IFG pars orbitalis or the anterior insula were significantly correlated with better therapy outcomes (Figure 1). Other classical demographic and lesion measures such as age and total lesion volume did not predict improvement in the naming test. For lesion volume, there was a subtlety: Wilmskoetter et al. (2022) found that average controllability of the IFG pars opercularis was correlated with total brain lesion volume and also with the lesion volume of the IFG pars opercularis itself. For the anterior insula, no correlation between regional or total brain lesion volume with modal controllability was found.

Using predictive statistical modeling, the authors then designed an optimal model and showed that, out of many potential candidate variables, only two variables were associated with improved therapy outcome. Specifically, one was average controllability of the IFG pars opercularis that together with IFG pars orbitalis, is part of Broca's region which is traditionally linked to language processing. The other variable was the Baseline Quotient of the Western Aphasia Battery, which measures baseline severity of aphasia.

## Implications for aphasia research

Given that language and speech are traditionally known to involve many cortical and subcortical areas both in healthy subjects (Poeppel, 2014) and post-stroke patients (Yourganov et al., 2016), the paper by Wilmskoetter et al. (2022) is particularly remarkable: It implies that a few specific controllability measures applied to a few selected brain regions are sufficient to predict naming score improvement after therapy. In view of recent studies and reviews on language recovery in aphasia (Kiran et al., 2019), it is not so much a surprise that IFG is involved, but that it seems to be involved nearly exclusively. Similar specialization applies to another network engaged in language comprehension, the so-called salience network, whose activity is correlated with residual language performance after a stroke (Brownsett et al., 2014). Naming test improvement was shown to be significantly correlated with connectivity strength of this network (Baliki et al., 2018). Here, Wilmskoetter et al. (2022) reported modal controllability of only the anterior insula, a part of the salience network, to be correlated with naming scores. This finding again highlights the importance of single regions within the larger language network in recovery from aphasia.

A further recent hypothesis in aphasia research is that language treatment outcome is affected by specific functional network connectivity patterns (Kiran et al., 2019), which can be easily obtained for a diverse group of post-stroke aphasia patients (Boyd et al., 2017). Baliki et al. (2018) suggested that global efficiency of functional connectivity, a measure of information integration in a network, is significantly correlated with language test score improvements. These findings are again largely in line with the results of Wilmskoetter et al. (2022), as average controllability describes efficiency of the brain network during state transitions.

Thus, a significant finding of the paper is that the post-stroke brain is theoretically controllable from single regions, as 68 of 70 individual connectomes examined by the authors were controllable. The other significant implication of this work is that there are specific topological measures, which can predict the therapy outcome. These measures can be obtained from the weighted adjacency matrices derived from individual connectomes. This suggests that targeting only a handful of brain regions, for example with transcranial direct current stimulation (Wortman-Jutt and Edwards, 2017) or transcranial magnetic stimulation (TMS; Naeser et al., 2012), might suffice for successful stroke recovery, which could open up the possibility for improved post-stroke aphasia rehabilitation therapies. Pursuing such a direction might be particularly promising since it was already shown in healthy subjects that controllability of the left IFG affected language performance under the influence of TMS (Medaglia et al., 2018, 2021). Concerning aphasia, several recent studies have shown that repetitive TMS (rTMS) applied to the contralesional IFG is advantageous for post-stroke recovery (Heikkinen et al., 2019; Kielar et al., 2022; Zumbansen et al., 2022).

## Discussion

Relying on the NCT framework, the results presented by Wilmskoetter et al. provide a comprehensive initial body of work showing which brain regions best predict post-stroke recovery from aphasia. At the same time, the interpretation of some of the results by Wilmskoetter et al. (2022) is not straightforward and some open questions remain. First of all, even though the controllabilities of IFG pars opercularis, IFG pars orbitalis and anterior insula are significant for the post-stroke language therapy outcome, they account only for 10, 12, and 12% of amount of variance explained by the partial regression model, respectively. The total amount of variance explained in the explanatory multivariable regression model is 31% for average controllability and 44% for modal controllability. At the same time, average controllability of IFG pars opercularis and baseline aphasia severity score still account only for 16% of variance explained in the predictive elastic net model (Wilmskoetter et al., 2022). These low amounts of variance explained might be due to the other language-related areas excluded from both models accounting for the rest of the variance such as Rolandic operculum, Heschl's gyrus and inferior parietal lobule, which are known to be associated with aphasia severity and subsequent recovery (Døli et al., 2021). Another reason for the low variance percentage being accounted for by the predictive elastic net model and the explanatory multivariable regression model might be the fact that the NCT framework is agnostic to many biological features of the brain, among those the directionality of the projection fibers and the non-linearity of brain dynamics. Moreover, the exclusion of some regions from the regression model due to their high multicollinearity might bias the regression outcome. The controllability of excluded regions might be correlated with the therapy outcome. This in turn might switch the main focus of the study, so that instead of one region being in control of the therapy outcome, a larger part of the language network could contribute significantly.

On the other hand, even if the brain may be theoretically controllable from a single region, the amount of energy needed to control it might be extremely large (Gu et al., 2015; Suweis et al., 2019). Also, Wilmskoetter et al. (2022) found that conventional local graph theory measures such as node strength and betweenness centrality were uncorrelated with controllability measures in post-stroke brains. This is surprising since Gu et al. (2015) reported a significant correlation between the node strength and controllability in the healthy brain. Thus, for further studies, it might be interesting to explore whether this divergence is due to the smaller sample size in the present study, potentially leading to a spurious lack of correlation, or whether it is an important feature distinguishing healthy and post-stroke connectomes.

For more definitive answers as to which specific topological properties of the residual language network after stroke underlie recovery from aphasia, a more comprehensive understanding than that provided by Wilmskoetter et al. is likely required. To start, the list of measures considered by Wilmskoetter et al., albeit large, is far from exhaustive. Further methods development could therefore add additional connectome measures to the modeling pipeline utilized by Wilmskoetter et al. (2022). Another possibility is to start looking into the controllability of homologous regions in the right hemisphere, especially after severe strokes and for chronic patients with aphasia (Kiran et al., 2019). A further example is to add classical topological quantities such as the clustering coefficient. Finally, the pipeline could benefit from local efficiency measures of white matter structural disconnections that were shown to provide explanatory power when assessing after-stroke dysfunctions (Griffis et al., 2019).

To conclude, the work by Wilmskoetter et al. (2022) suggests that the post-stroke brain is theoretically controllable and that the controllability properties of single brain regions, particularly IFG pars opercularis and anterior insula, can be potential biomarkers for predicting post-stroke aphasia recovery. This finding might be foundational for understanding mechanisms underlying aphasia and it opens an avenue for exploring new stroke rehabilitation strategies. These strategies could aim at manipulating controllability, in the sense of NCT, of the brain regions of interest. To achieve this, it will be crucial to extend the results of Wilmskoetter et al. (2022) and understand which changes in structural connectivity underlie different dynamical regimes and lead to changes in controllability of the post-aphasic brain.

## Author contributions

MP wrote the original draft of this opinion manuscript. All authors contributed to its conception and development, revised, and approved the final version.

## Funding

MP was funded by the Deutsche Forschungsgemeinschaft (DFG, German Research Foundation) - SFB 936 - Project-ID 178316478-A1/Z3. KF was funded by the DFG - SFB 936 - Project-ID 178316478-A1. WB was funded by the DFG – Project-ID 434434223–SFB 1461.

## Conflict of interest

The authors declare that the research was conducted in the absence of any commercial or financial relationships that could be construed as a potential conflict of interest.

## Publisher's note

All claims expressed in this article are solely those of the authors and do not necessarily represent those of their affiliated organizations, or those of the publisher, the editors and the reviewers. Any product that may be evaluated in this article, or claim that may be made by its manufacturer, is not guaranteed or endorsed by the publisher.
